# Multilocus sequence typing of *Campylobacter concisus* from Danish diarrheic patients

**DOI:** 10.1186/s13099-016-0126-0

**Published:** 2016-09-22

**Authors:** Hans Linde Nielsen, Henrik Nielsen, Mia Torpdahl

**Affiliations:** 1 Department of Clinical Microbiology, Aalborg University Hospital, Aalborg, Denmark; 2 Department of Infectious Diseases, Aalborg University Hospital, Aalborg, Denmark; 3 Department of Clinical Medicine, Aalborg University, Aalborg, Denmark; 4 Department of Microbiology and Infection Control, Statens Serum Institut (SSI), Copenhagen, Denmark

**Keywords:** *Campylobacter concisus*, MLST, Diarrhea, Gastroenteritis, Sequencing, Emerging, 23S, Genomospecies, Genetic diversity, Collagenous colitis, Crohn’s disease

## Abstract

The emerging enteric pathogen *Campylobacter concisus* is associated with prolonged diarrhea and inflammatory bowel disease. Previous studies have shown that *C. concisus* strains are very genetically diverse. Nevertheless, *C. concisus* strains have been divided into two genomospecies, where GS1 strains have been isolated predominantly from healthy individuals, while the GS2 cluster consists of isolates primarily from diarrheic individuals. The aim of the present study was to determine the genetic diversity of *C. concisus* isolates from Danish diarrheic patients. Multilocus sequence typing using the loci *aspA*, *atpA*, *glnA*, *gltA*, *glyA*, *ilvD* and *pgm*, as well as genomospecies based on specific differences in the 23S rRNA, was used to characterize 67 isolates (63 fecal and 4 oral), from 49 patients with different clinical presentations (29 with diarrhea, eight with bloody diarrhea, seven with collagenous colitis and five with Crohn’s disease). MLST revealed a high diversity of *C. concisus* with 53 sequence types (STs), of which 52 were identified as ‘new’ STs. Allele sequences showed more than 90 % similarity between isolates, with only four outliers. Dendrogram profiles of each allele showed a division into two groups, which more or less correlated with genomospecies A and genomospecies B. However, in contrary to previous results, this subgrouping had no association to the clinical severity of disease.

**Electronic supplementary material:**

The online version of this article (doi:10.1186/s13099-016-0126-0) contains supplementary material, which is available to authorized users.

## Background

*Campylobacter jejuni* is the most commonly reported bacteriologic agent in gastrointestinal infectious disease [1]. A related species, *Campylobacter concisus,* was first isolated from human periodontal lesions [2], and is now considered part of the normal human oral microbiota [3]. However, in a recent population based study from Denmark, *C. concisus* was the most prevalent *Campylobacter* species in diarrheic stool samples, by cultivation using mCCDA plates as well as a polycarbonate filter technique on blood agar plates [4].

Recent studies have associated *C. concisus* to prolonged gastroenteritis in children as well as adults [5, 6]. Interestingly, *C. concisus* infection appears to cause milder, more prolonged diarrhea compared to *C. jejuni* infection, while *C. concisus* positive patients still present with the same gastrointestinal complaints following acute gastroenteritis as *C. jejuni* positive patients [6]. Furthermore, several studies have shown a high prevalence of *C. concisus* DNA in mucosal biopsies from patients with inflammatory bowel disease (IBD) [7–10] and one study showed a possible association between *C. concisus* infection and microscopic colitis, especially collagenous colitis [6].

Previous studies have shown that *C. concisus* is genetically diverse and have described two major clusters or genomospecies (GS), based on both amplified fragment length polymorphism (AFLP) and strain typing using 23S rRNA PCR [11–14]. By use of MLST, Miller et al. [15] also showed that strains of *C. concisus* could be grouped into two clusters that corresponded to GS A and GS B, however, they did not analyze correlation with different disease presentations. In addition, Mahendran et al. by use of six housekeeping genes [16], showed that oral *C. concisus* strains also comprised of two GS. Finally, Deshpande et al. sequenced eight strains of *C. concisus* and showed that *C. concisus* strains were highly genetically diverse [17, 18].

The aim of the present study was to determine the diversity of *C. concisus* isolates from Danish diarrheic patients by use of the MLST scheme by Miller et al. [15], and to compare GS with the clinical presentation of disease [6].

## Methods

Sixty-seven isolates (63 fecal and 4 oral), from 49 patients (29 females/20 males) with different clinical presentations were analyzed. Twenty-nine with diarrhea (age range: 0–86 years), eight with bloody diarrhea (0–78 years), seven with collagenous colitis (53–80 years), and five with Crohn’s disease (age 2–73 years) were selected for the study.

All strains were isolated at the Department of Clinical Microbiology, Aalborg University Hospital, Denmark and MLST was performed at SSI. *C. concisus* was isolated using the filter technique on 5 % horse blood agar plates, containing 1 % yeast extract (SSI Diagnostica, Hillerød, Denmark), and incubated at 37 °C in a microaerobic atmosphere with 3 % hydrogen. Final identification was obtained through a species-specific real-time PCR based on the cpn60 gene, as described elsewhere [19], as well as MALDI-TOF analysis (BRUKER DALTONIK GmbH, Bremen, Germany) [20]. All strains were stored in nutrient beef broth with 10 % glycerine (SSI Diagnostica, Hillerød, Denmark) at −80 °C until use.

Isolates were re-cultivated as described above and DNA was extracted using an on-board protocol with the NucliSENS® easyMAG® platform (BioMérieux, Marcy-l’Étoile, France). PCR amplification of the 23S rRNA gene was conducted by use of one forward primer (MUC1) and two reverse primers (CON1 and CON2) used independently, and amplified PCR products were visualized on QiAXcel Advanced screen gel for verification of product size (Qiagen, Hilden, Germany). Isolates amplifying with either MUC1/CON1 or MUC1/CON2 primers were assigned to GS A or B, respectively, according to Kalischuk and Inglis [14].

Seven housekeeping genes (*aspA*, *atpA*, *glnA*, *gltA*, *glyA*, *ilvD* and *pgm*) were amplified from the 67 isolates using the MLST scheme of Miller et al. [15]. All housekeeping genes were sequenced using both forward and reverse primers and all sequences were evaluated manually. Sequences with low-quality bases in the chromatograms were re-sequenced to give high-quality bases in the chromatograms. BioNumerics version 7.1 (Applied Maths NV, Sint-Martens-Latem, Belgium) was used for sequence analysis and further used to generate an UPGMA tree. All sequences were submitted to the pubmlst.org database and published online (http://pubmlst.org/campylobacter/).

## Results

MLST revealed a high diversity of *C. concisus* with 53 sequence types (STs), of which 52 were identified as ‘new’ STs. The full list of *C. concisus* strains used in this study is available in the Additional file 1. The high degree of variation across the *C. concisus* STs was reflected by the large number of alleles within the collection of 67 isolates. The number of alleles were as follows: *aspA*: 39, *atpA*: 34, *glnA*: 37, *gltA*: 36, *glyA*: 39, *ilvD*: 39, *pgm*: 45.

**Fig. 1:**
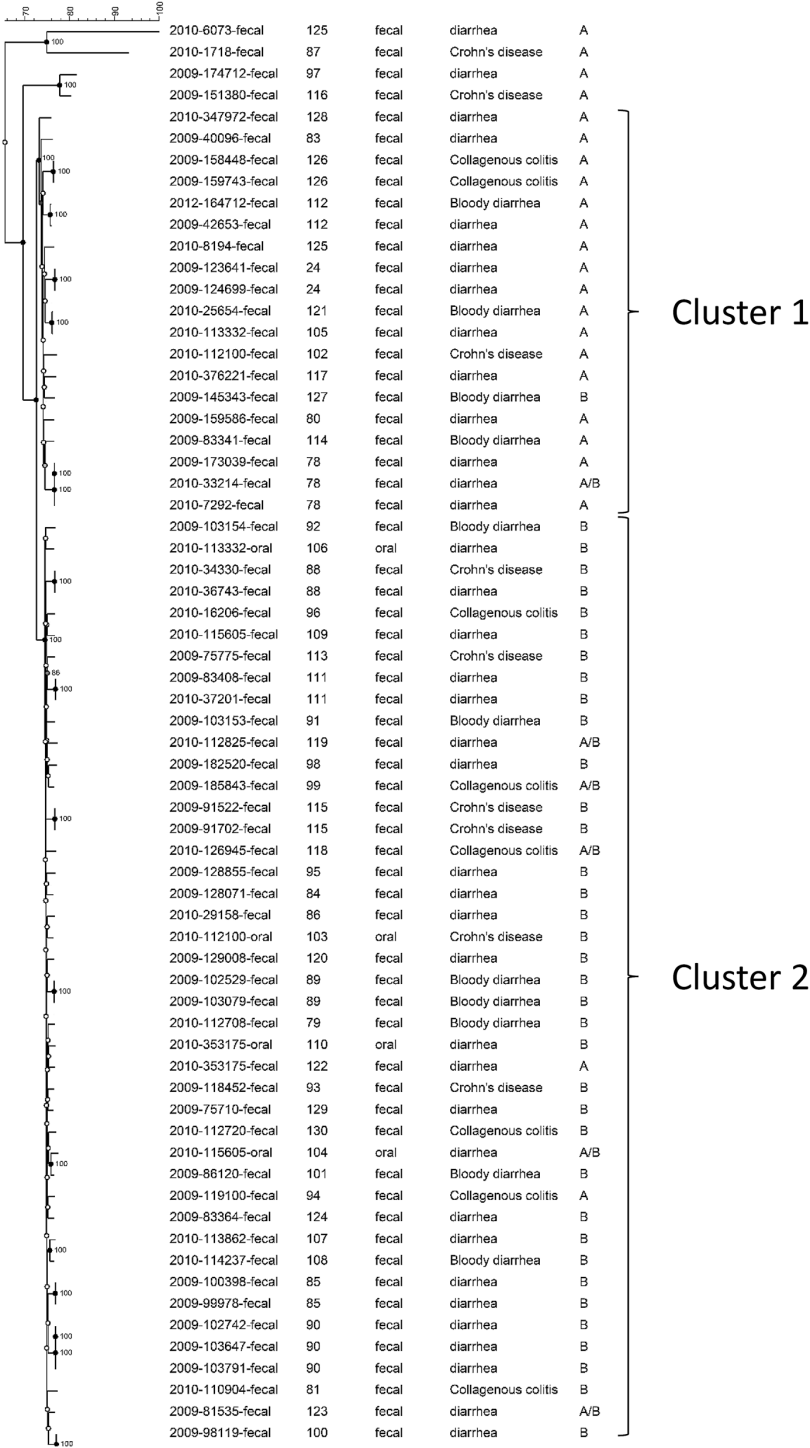
Neighbour joining tree based on merged sequences of the seven housekeeping genes for the *C. concisus* MLST scheme. ST number, source of the *C. concisus* strain, the clinical presentation and genomospecies (A, B or A/B) are shown

**Fig. 2:**
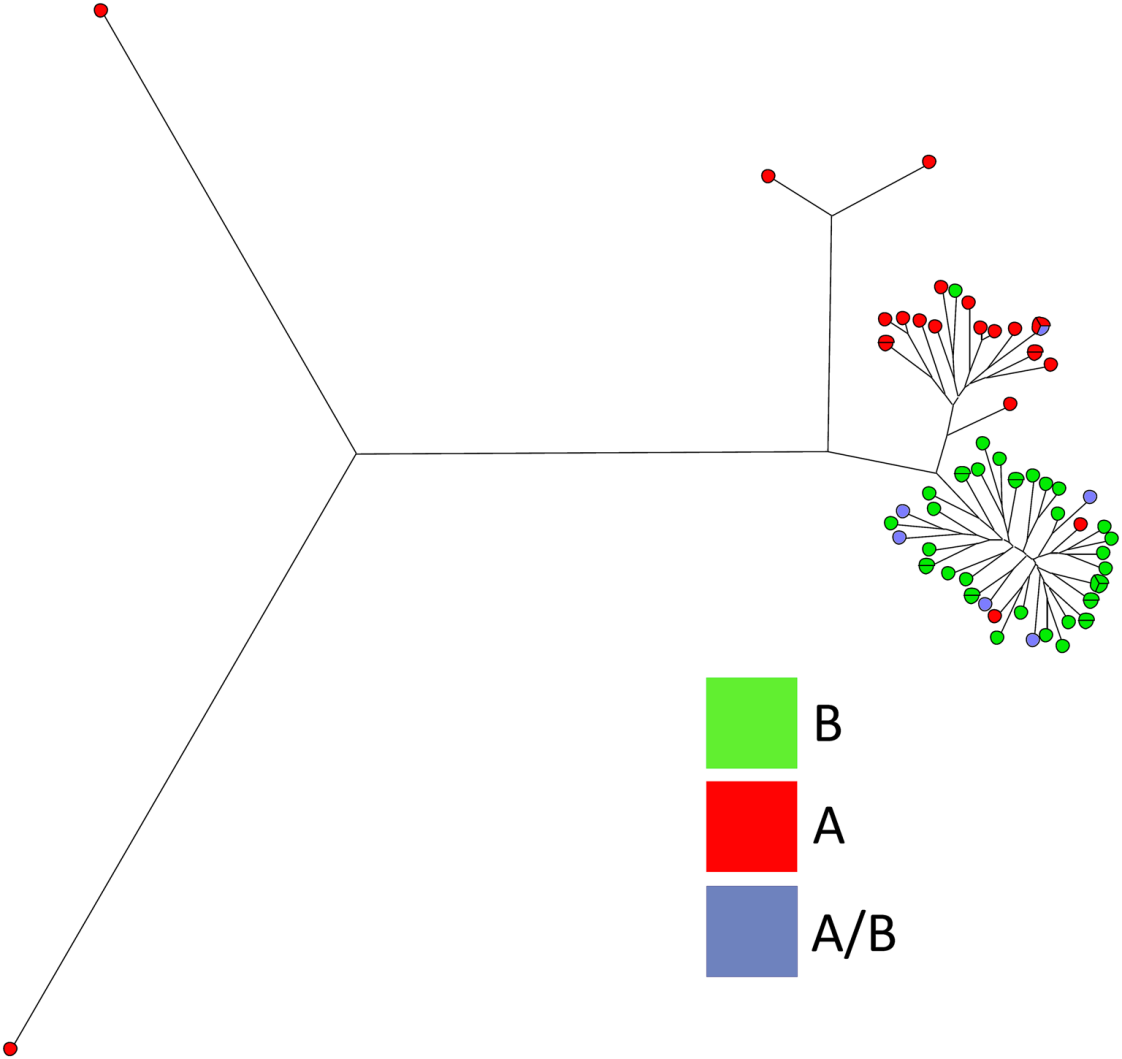
Maximum parsimony tree using concatenated sequences merging of the seven housekeeping genes. The *colors* represent genomospecies (*A*, *B* or *A/B*) showing a clear division into two main clusters with four outliers

We found 23 and 38 strains positive only for GS A and GS B, respectively, while six strains were PCR positive for both GS A and GS B. A phylogenetic tree was generated based on the concatenated sequences of the seven housekeeping genes (3345 bp). With the exception of four outliers, isolates were divided into two major clusters (cluster 1 and 2) where all sequences displayed more than 90 % similarity (Fig. 1). The correlation between 23S and MLST was high and almost all strains in cluster 1 were GS A whereas GS B were found in cluster 2 (Figs. 1, 2). The subgrouping had no significant association with clinical disease, since all the different clinical presentations (diarrhea, bloody diarrhea, collagenous colitis, and Crohn’s disease) were distributed within the two clusters (Fig. 1).

## Discussion

In this study, we found that *C. concisus* isolates from Danish diarrheic patients were highly genetically diverse by use of the MLST-scheme according to Miller et al., an accurate tool for *C. concisus* genomotyping [15]. We combined the MLST method with 23S and confirmed previous results with division into two groups, almost similar to GS A (cluster 1) and GS B (cluster 2), but found no association with clinical presentation.

We were unable to include fecal isolates from healthy individuals, but our GS A isolates were from patients with different clinical presentations. Previously, Kalischuk and Inglis included five *C. concisus* isolated from healthy human feces, and four of these isolates were positive for GS A [14]. Those isolates exhibited increased hemolytic ability, apoptotic DNA fragmentation, and IL-8 induction, whereas ALFP cluster 2 isolates, mainly positive for GS B, were predominantly from diarrheic patients, exhibiting higher levels of epithelial invasion and translocation, consistent with factors causing diarrheal disease.

We included four oral strains isolated from patients with clinical disease and not surprisingly, they all had different *C. concisus* isolates in stools. One of the four oral strains was both GS A and GS B positive and the remaining all belonged to cluster 2 (GS B positive), in accordance with Kalischuk and Inglis, suggesting GS2 to have a higher potential for diarrheic disease [14]. Previous data has shown that both oral and enteric isolates of *C. concisus* can cause epithelial barrier dysfunction and apoptosis [21], therefore suggesting that *C. concisus* strains colonizing the intestinal tract originate from oral *C. concisus* strains [16, 22].

Although, our result showed no significant association with clinical presentation between the two groups, previous findings, as mentioned above, have shown a higher pathogenic potential in GS B positive isolates [14]. In one study, four enteric strains isolated from patients with bloody diarrhea all belonged to AFLP cluster 2 (i.e., genomospecies 2), correlating to GS B [12]. Another study supported this finding, showing that although enteric isolates in general did not form distinct clusters, oral and enteric isolates that were been proven invasive, belonged to the same cluster [16]. We included eight patients with bloody diarrhea of which four strains were in cluster 1, according to the MLST-scheme, however, one of these (ST 127) was positive for GS B instead of GS A. There were also isolates from patients with collagenous colitis and Crohn’s disease in cluster 1, but numbers were limited.

Many molecular sub typing methods have been developed to characterize *Campylobacter* species, but only a few are commonly used in molecular epidemiology studies. *C. concisus* has been isolated in high numbers from Danish diarrheic stool samples, and while MLST is suitable for data exchange between different laboratories, it may not be applicable for *C. concisus,* because the number of STs is very high. Twelve ST-types from 26 strains were represented more than once. However, 17/26 (ST: 24, 85, 89, 90, 100, 115, 125 and 126) were strains that were isolated twice (one triplicate), from patients within the same diarrheic episode and with a minimum of time-span between stool collection. The remaining nine strains (ST: 78, 88, 111 and 112) were isolated from different patients with no obvious relatedness.

A recent study by Kirk et al. [10] showed a relative high isolation rate of *C. concisus* from saliva samples, intestinal biopsies and stools from both IBD patients and healthy controls. It would be highly interesting to see whether these patients were infected or colonized with different strains, for example by use of the MLST-scheme or by whole genome sequencing technologies.

In conclusion, we have shown, in accordance with previous studies, that *C. concisus* strains from Danish diarrheic patients are highly genetically diverse. A sequence-based phylogeny resulted in division of the strains into two major groups, almost similar to GS A and GS B, but we found no association with clinical presentation.

## Additional file

10.1186/s13099-016-0126-0 All isolates.
