# Supplementary material for: Multilocus sequence typing of Campylobacter concisus from Danish diarrheic patients
Source: Gut Pathog. 2016 Sep 22;8:44. doi: 10.1186/s13099-016-0126-0 (PMC5034547; doi:10.1186/s13099-016-0126-0)
Supplement: Supplementary file 1 — 10.1186/s13099-016-0126-0 All isolates. [file 13099_2016_126_MOESM1_ESM.pdf]

Patient ID	CMA-Key	MLST ST	Source	Age	Sex	Clinical presentation	Genomospecies
1	2010-112100-oral	103	oral	36	Female	Crohn's disease	B
1	2010-112100-fecal	102	fecal				A
2	2010-113332-oral	106	oral	57	Female	diarrhea	B
2	2010-113332-fecal	105	fecal				A
3	2010-115605-fecal	109	fecal	42	Male	diarrhea	B
3	2010-115605-oral	104	oral				A/B
4	2010-353175-fecal	122	fecal	35	Male	diarrhea	A
4	2010-353175-oral	110	oral				B
5	2009-91522-fecal	115	fecal	29	Male	Crohn's disease	B
5	2009-75775-fecal	113	fecal				B
5	2009-91702-fecal	115	fecal				B
6	2009-98119-fecal	100	fecal	71	Female	diarrhea	B
6	2009-98127-fecal	100	fecal				B
7	2009-100398-fecal	85	fecal	78	Female	diarrhea	B
7	2009-99978-fecal	85	fecal				B
8	2009-102529-fecal	89	fecal	46	Male	Bloody diarrhea	B
8	2009-103079-fecal	89	fecal				B
9	2009-102742-fecal	90	fecal	76	Female	diarrhea	B
9	2009-103647-fecal	90	fecal				B
9	2009-103791-fecal	90	fecal				B
10	2009-103153-fecal	91	fecal	1	Female	Bloody diarrhea	B
10	2009-103154-fecal	92	fecal				B
11	2009-123641-fecal	24	fecal	80	Male	diarrhea	A
11	2009-124699-fecal	24	fecal				A
12	2009-128071-fecal	84	fecal	18	Female	diarrhea	B
12	2009-128855-fecal	95	fecal				B
13	2009-158448-fecal	126	fecal	80	Male	Collagenous colitis	A
13	2009-159743-fecal	126	fecal				A
14	2010-1718-fecal	87	fecal	2	Female	Crohn's disease	A
14	2010-34330-fecal	88	fecal				B
15	2010-6073-fecal	125	fecal	73	Male	diarrhea	A
15	2010-8194-fecal	125	fecal				A
16	2012-164712-fecal	112	fecal	73	Female	Bloody diarrhea	A
16	2010-25654-fecal	121	fecal				A
17	2010-126945-fecal	118	fecal	65	Female	Collagenous colitis	A/B
18	2010-112825-fecal	119	fecal	40	Female	diarrhea	A/B
19	2010-110904-fecal	81	fecal	75	Female	Collagenous colitis	B
20	2009-83364-fecal	124	fecal	59	Male	diarrhea	B
21	2009-174712-fecal	97	fecal	45	Male	diarrhea	A
22	2009-145343-fecal	127	fecal	77	Female	Bloody diarrhea	B
23	2009-118452-fecal	93	fecal	19	Female	Crohn's disease	B
24	2009-83408-fecal	111	fecal	1	Male	diarrhea	B
25	2010-37201-fecal	111	fecal	1	Female	diarrhea	B
26	2009-42653-fecal	112	fecal	1	Female	diarrhea	A
27	2009-40096-fecal	83	fecal	8	Male	diarrhea	A
28	2009-75710-fecal	129	fecal	0	Male	diarrhea	B

29	2009-119100-fecal	94	fecal	63	Female	Collagenous colitis	A
30	2009-83341-fecal	114	fecal	78	Female	Bloody diarrhea	A
31	2009-173039-fecal	78	fecal	56	Female	diarrhea	A
32	2010-33214-fecal	78	fecal	59	Male	diarrhea	A/B
33	2010-7292-fecal	78	fecal	83	Male	diarrhea	A
34	2010-36743-fecal	88	fecal	1	Male	diarrhea	B
35	2009-81535-fecal	123	fecal	2	Male	diarrhea	A/B
36	2010-16206-fecal	96	fecal	69	Female	Collagenous colitis	B
37	2010-29158-fecal	86	fecal	70	Female	diarrhea	B
38	2009-151380-fecal	116	fecal	73	Male	Crohn's disease	A
39	2010-376221-fecal	117	fecal	58	Male	diarrhea	A
40	2010-112708-fecal	79	fecal	0	Female	Bloody diarrhea	B
41	2009-182520-fecal	98	fecal	7	Male	diarrhea	B
42	2010-114237-fecal	108	fecal	59	Female	Bloody diarrhea	B
43	2010-112720-fecal	130	fecal	53	Female	Collagenous colitis	B
44	2009-159586-fecal	80	fecal	32	Female	diarrhea	A
45	2009-86120-fecal	101	fecal	59	Female	Bloody diarrhea	B
46	2010-347972-fecal	128	fecal	38	Male	diarrhea	A
47	2010-113862-fecal	107	fecal	64	Female	diarrhea	B
48	2009-129008-fecal	120	fecal	86	Female	diarrhea	B
49	2009-185843-fecal	99	fecal	80	Female	Collagenous colitis	A/B
